# Utilization and determinants of maternity waiting homes among pastoralist mothers in Dire district, southern Ethiopia: a mixed-methods study

**DOI:** 10.3389/fgwh.2024.1446500

**Published:** 2024-10-25

**Authors:** Hassan Mahamad Duba, Mulugeta Mekuria, Erean Shigign Malka, Addisu Waleligne Tadesse, Ketema Gashaw, Ketema Eshetu

**Affiliations:** Department of Public Health, College of Health Sciences, Salale University, Fiche, Ethiopia

**Keywords:** maternity waiting home, utilization, determinants, pastoralist, mothers, Ethiopia

## Abstract

**Background:**

Maternity waiting homes are cost-effective, World Health Organization-approved components of comprehensive prenatal, delivery, and postpartum care strategies. However, few community-based studies within Ethiopia's pastoralist communities, and none in the study area, have been conducted to determine actual usage or to gain a thorough understanding of the factors influencing utilization.

**Methods:**

A cross-sectional study, supplemented by qualitative methods, was conducted from June 25 to July 25, 2023. A simple random sampling technique was used to select 305 study participants. Data were gathered through an interviewer-administered questionnaire, entered into Epi-data version 3.1, and analyzed using SPSS version 25. Descriptive data were presented in tables, graphs, text, and percentages. Bivariate logistic regression identified candidate predictors at a *P*-value of <0.25, and predictors of maternity waiting home utilization were identified through multivariate logistic regression at a 95% confidence interval and *P*-value of <0.05. Qualitative interviews were transcribed, translated, and thematically analyzed.

**Results:**

The prevalence of maternity waiting home use in the study area was 35.2% (95% CI: 30.9%, 39.5%). Factors associated with maternity waiting home utilization included travel time greater than 60 min to nearby health facilities (AOR: 5.47 CI: 1.77, 16.91), good knowledge of danger signs of pregnancy (AOR: 5.41, CI: 1.86, 15.79), lack of a caretaker to household tasks (AOR: 0.1, CI: 0.03, 0.31), and a refusal to accept a waiting time of 2–4 weeks (AOR: 0.24 CI: 0.08, 0.74). The qualitative findings underscored hurdles such as resource constraints, challenges in providing maternity services, and the importance of community awareness and access to network connectivity in ensuring safe childbirth.

**Conclusion:**

This study aims to determine the utilization of maternity waiting homes and the factors associated with their use among women who gave birth within the last year in the Dire district, Borana zone, southern Ethiopia. The prevalence of maternity waiting home use was low compared to national efforts to promote this service. Longer travel time, lack of a caretaker, good knowledge of danger signs of pregnancy, and a refusal to accept a waiting time of 2–4 weeks were associated with maternity waiting home use in this study.

## 1 Introduction

In 2020, globally, one preventable maternal death related to pregnancy occurs every two minutes, with the majority happening in rural areas, underdeveloped regions, and among pregnant teenage girls (1). Sub-Saharan Africa alone accounts for approximately 70% of global maternal deaths, followed by Central and Southern Asia, which accounts for almost 17% (2). About 66% of these global fatalities, with half occurring in developing nations, result directly from unsafe practices during childbirth (3–5). In contrast, maternity waiting homes have had a significant impact on reducing prenatal asphyxia, newborn mortality, and maternal deaths by facilitating skilled delivery and bridging the urban-rural disparity (6–8). Recognizing the significance of these advancements, a recommendation was issued in the late 20th century.

Maternity waiting homes were defined in the 1990s by the World Health Organization (WHO) as “residential facilities near a qualified medical facility where “high-risk” women can wait for their delivery and be transferred to a nearby medical facility shortly before delivery” (9, 10). Hence, they are advisable for low- and middle-income countries where the bulk of births take place at home and maternal mortality is high. However, despite their vital role in enhancing the well-being of mothers and children, maternity waiting homes remain underused for various reasons. Previous studies identified factors such as advanced age, pregnancy difficulties, a history of caesarean section (C/S), and regular antenatal care (ANC) visits as crucial factors that enhance usage (11–13). However, qualitative research revealed that culturally unacceptable care, an awareness gap about maternity waiting home, a lack of caretaker-at-home work, and service-related fees were obstacles to its use (14, 15). Furthermore, a study in Indonesia found a low use rate of 18.7%, with financial barriers being the primary reason for non-use (16).

The Ethiopian Federal Ministry of Health (FMOH) launched the Health Sector Transformation Plan (HSTP) II, aiming to reduce the maternal mortality ratio (MMR) from 401 per 100,000 live births to 279 by 2024/25, with an emphasis on promoting maternity waiting homes as a key initiative (17). Since 2014, numerous healthcare facilities have instituted their own maternity waiting areas (18), despite significant disparities in utilization observed across districts or regions within the country. Notably, the Southern region exhibits a wide range of usage rates, from 16.7% at Jinka zonal hospital (19) to a substantially higher 67.8% in the Sidama zone (20). Similarly, in the Amhara region, rates range from 16.2% in Dabat district (11) to 20.5% in the East Belesa districts (21). Oromia also shows a marked disparity, with usage rates ranging from 7% in the Jimma zone (22) to a 34.0% in the Finfinnee special zone (23).

Consequently, the national institutional delivery rate is at 48% (24), dropping to 41% in the Oromia regional state (25), revealing significant disparities across various societal contexts, particularly among agrarian and pastoralist communities. For instance, there is considerable variation in institutional delivery, ranging from 13.9% in the Liben district of pastoralist settings (26) to 64% in the Ambo district of farming settlements (27). This demonstrates that the majority of mothers in a pastoralist area have been delivering at home, exposing them to a high risk of maternal death. In the Borana pastoralist communities, mothers who gave birth at home were twice as likely to die as mothers who gave birth at a health facility, and 86% of the fatalities were due to direct obstetric causes, with hemorrhage accounting for 45% of the deaths (28).

Hence, in pastoral communities where most maternal deaths stem from direct childbirth complications and healthcare access is limited, maternity waiting home emerges as one of the most impactful and efficient solutions available. However, most research in this context was done at the facility level, evaluating the intentions of pregnant women to use maternity waiting home (12, 29–31). In addition, to the researcher's knowledge, there was limited investigation of community-based studies addressing the actual usage and underlying factors influencing the utilization of maternity waiting homes in Ethiopia. Furthermore, previous studies have largely overlooked the marginalized pastoralist communities with mobile lifestyles. Thus, this study aimed to determine the prevalence and factors influencing maternity waiting home use among pastoralist mothers who gave birth in the past year in the Dire district, Borana zone, Oromia regional state, southern Ethiopia.

## 2 Methods and materials

### 2.1 Study area and period

**Figure 1:**
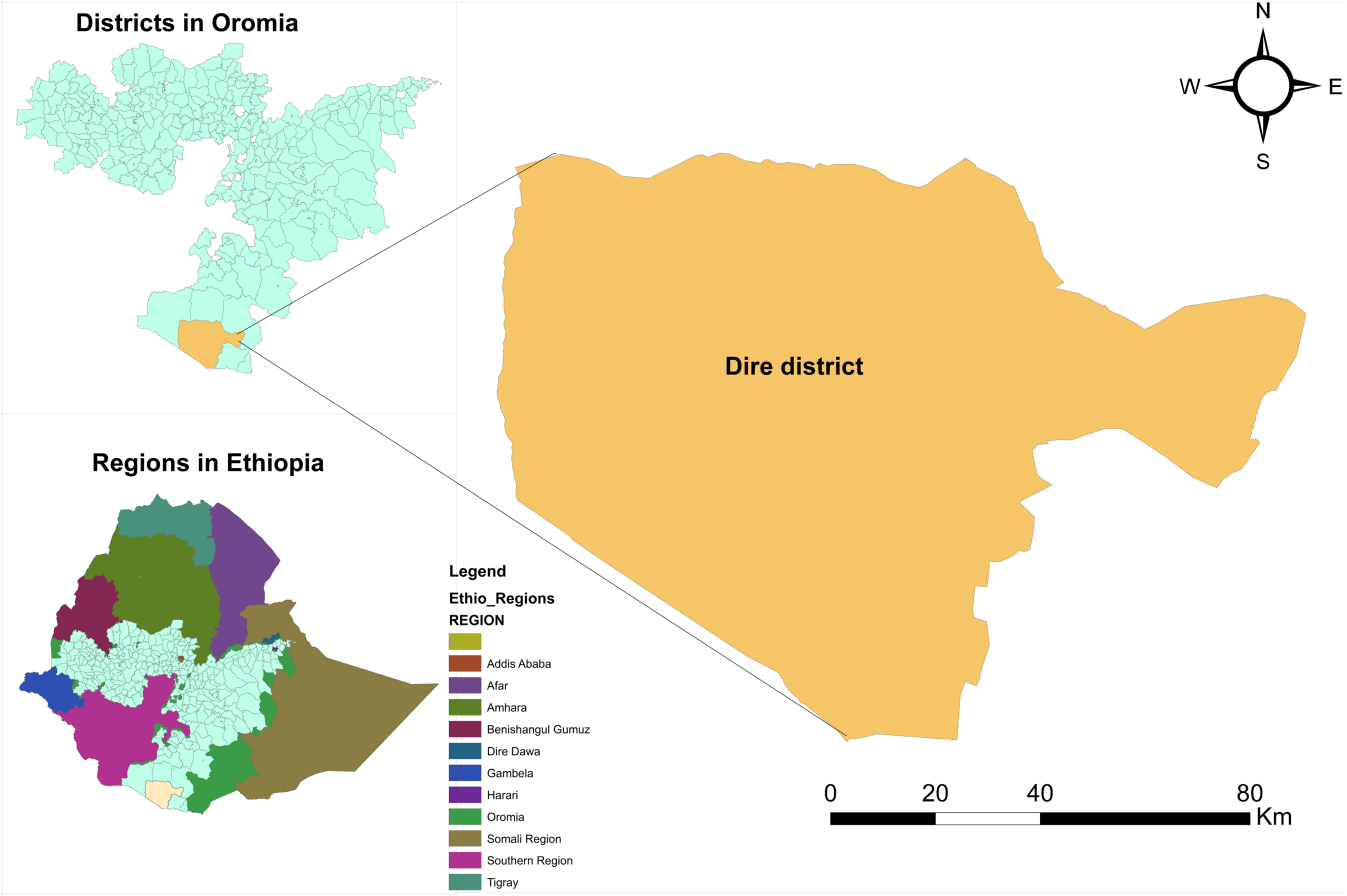
Map showing the study district in the Borana zone, Oromia regional state, Ethiopia.

The study was conducted in Dire districts, Borana zone, Oromia regional state, southern Ethiopia. Mega is a town in the Dire district, 666 kilometers’ south of Addis Ababa on the main road to Moyale. Lowland topography and an arid climate characterize the area. Residents are pastoralists who primarily depend on raising livestock for their subsistence. Dire district has 19 kebeles (subdistricts). Five lower clinics, two medium clinics, and drug stores make up the private health facilities, while there is one district hospital, three health centers, and sixteen rural health posts in the public sector. According to Woreda-Based Health Sector Plan (WBHSP) projections from the 2007 Ethiopian census, Dire district has a total population of 55,847 and 11,635 households, with 12,976 urban residents, 42,871 rural residents, 1,938 estimated deliveries, and 12,359 women of reproductive age in 2022/2023 (32). This study was conducted from June 25 to July 25, 2023. The study area map is shown below (Figure 1).

### 2.2 Study design and population

This study employed a community-based cross-sectional design, complemented by qualitative methods to enhance the depth of the findings. The source population included all women who gave birth in the Dire district during the previous year. The study population focused on women who had given birth within that year and were living in selected kebeles of the district during data collection. Additionally, interviews with key informants from the health office and the community were conducted to further enrich the data. However, women who were critically ill and unable to participate during the data collection period were excluded.

### 2.3 Sample size determination and sampling procedure

**Figure 2:**
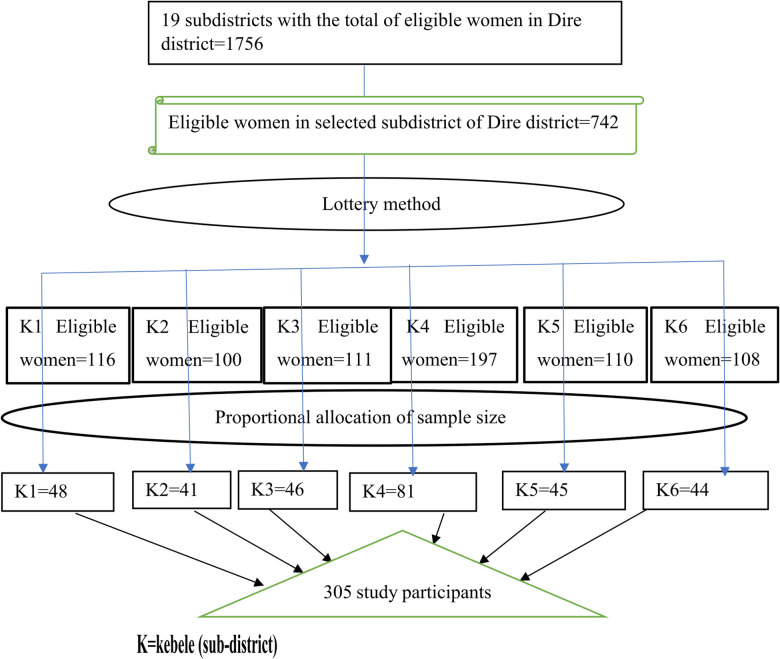
Schematic presentation of sampling procedure among women who gave birth in the last year in the Dire district, southern Ethiopia, 2022/2023.

For the quantitative part, after reviewing various similar studies, the 23.6% proportion of maternity waiting home utilization from the Digelu and Tijo district study with the largest sample size was chosen (33). The sample size was then calculated manually, using a single population proportion with the assumption of a 95% confidence interval and a decision precision of 5%, as follows:

$$n=\frac{{\left(z\alpha /2\right)}^{2}\;p(1-p)}{d^{2}\;}=\frac{{\left(1.96\right)}^{2}(0.236)(0.764)}{0.05^{2}}=277,$$

where *n* is the required sample size, *z* is the critical value of the 95% confidence interval under the normal distribution curve (1.96), *α* is the level of significance, *p* is the percentage of maternity waiting home users, 1 − *P* is the percentage of non-users, and d is decision precision. So, by adding 10% of the non-respondent rate, the final sample size was 305. The sample size of the second objective was also calculated using Epi-info version 7.2.5.0 with the assumptions of a 95% confidence interval and 80% power, and finally, the largest sample size (i.e., 305) was taken. Dire district has 19 subdistricts, of which six were selected using simple random sampling techniques (lottery methods). Of the 1,756 women who gave birth in the district last year, 742 were in the selected subdistricts. A proportional allocation was made to the selected subdistricts based on the number of women who gave birth. Then, a simple random sampling technique (random number generator) was used to select study participants using the information in the pregnant women's registration book as a sampling frame (Figure 2).

#### 2.3.1 For the qualitative investigation

Six key informant interviews (KIIs), which included three health office representatives, two leaders of the Women Development Army (WDA), and one elder (a religious leader), were purposively chosen.

### 2.4 Study variables

Maternity waiting-home utilization was a dependent variable. The independent variables were socio-demographic factors (age, marital status, educational level, family income, and occupation), knowledge of danger signs of pregnancy, attitude towards care, respect, compassion (CRC) of health professionals and maternity waiting home use, and obstetric and facility-related variables.

### 2.5 Measurements and operational definitions

#### 2.5.1 Maternity waiting home utilization

In the context of this study, a pregnant mother was designated as a “user” if she stayed at a maternity waiting home within the study area while awaiting labor as she neared the term of her most recent delivery, preceding the onset of labor, regardless of how long she stayed. It was measured by asking, “ Did you stay at the maternity waiting home just prior to onset of labor during your most recent delivery?” with a “yes” or “no” response (18, 34).

#### 2.5.2 Past experience of maternity waiting home utilization

It is the use of maternity waiting home before the most recent intake. Evaluated by answering “yes” or “no” to the question, “Have you ever used a maternity waiting home before current use?”.

#### 2.5.3 Household income

A principal component analysis was used to create the household wealth index based on information on asset ownership, the number of animals owned, owning a mobile phone, owning a motor bicycle, electricity supply to the home, health insurance, drinking water source, type of toilet, and type of materials used for house floor construction. Eventually, the wealth index was divided into three categories: poor, medium, and rich. According to the economic status variable, the lowest 33% of families were classified as poor, the top 33% as rich, and the remainder as medium (22, 23).

#### 2.5.4 Knowledge of the danger signs of pregnancy

Those who listed three or more pregnancy danger signs, such as vaginal bleeding, gush of fluid per vagina, severe abdominal pain, high-grade fever, fainting, decreased fetal movement, blurred vision, severe headache, oedema, or body swelling, were classified as having good knowledge (11, 35).

#### 2.5.5 Attitude towards maternity waiting home usage

Eight questions on a five-point Likert scale were used to examine mothers’ views, feelings, and ideas about maternity waiting home utilization. The replies were then summed up, and each respondent's total score was obtained. Lastly, the mean score was determined, and those who scored equal to or more than the mean were classified as having a “more positive attitude” (36).

### 2.6 Data collection tools, procedure, and quality control

#### 2.6.1 For quantitative

Data were collected in a face-to-face, household-level interview using structured questionnaires developed after reviewing relevant literature (11, 20, 33, 37–39). The questionnaire included information regarding sociodemographic, obstetrics, service-related characteristics, and knowledge and attitudes of mothers. The instrument was written in English, translated into Afan Oromo, the local language of the study area, and then back translated into English to ensure consistency. To ensure data quality, three health extension workers (HEWs) were recruited as data collectors, and one health officer, all fluent in the local languages, was recruited as a supervisor. They were trained for two days before data collection. A questionnaire pre-test was conducted on 5% of the total sample size, which was 15 women from non-selected subdistricts but had a similar population. Based on pre-test experiences, modifications to the arrangement of tools, merging related questions, and clarifying unclear questions for actual data collection were made. Cronbach's alpha was used to verify tool internal consistency for knowledge and attitude questions, and a value of >0.85 was considered good enough. During the data collection phase, the researchers and supervisors closely monitored the process, ensuring correctness, and promptly made any necessary corrections to maintain standards.

After the data were collected, editing, checking, and processing for completeness and consistency were done to ensure that the information had been properly collected and recorded. To reduce errors, double entries by two computers simultaneously using a pre-prepared and adjusted template of Epi-data version 3.1 were made. Data were coded using non-overlapping numerical or letter codes to distinguish between answers and missing values. The data cleaning process was also performed to ensure that data entered into a computer was identical to data on paper by looking for consistency of values, providing a good skipping pattern, and controlling for what must be entered to have more quality data for analysis. After cleaning, the data were sorted using simple frequency and tabulating variables for consistency. As a result, any differences, outliers, or missing values were corrected. Finally, data validation was performed after cleansing and before analysis to ensure the maximum optimal quality level of the data.

#### 2.6.2 For qualitative

Data were collected by the local language (Afan Oromo) version of the semi-structured KII guide. The guide was first developed in English by the investigator, translated into Afan Oromo, and checked for clarity by experts. The guide explored maternity waiting home awareness, health professional behavior, maternity waiting home acceptability, barriers and facilitators of service usage, and probs follow-up at each discussion point. One master's degree holder facilitated KII, and during an interview, backup notes and audio recordings were recorded. Each discussion took 60–90 min.

Quality assurance of qualitative was also maintained by four dimension criteria adopted from Lincoln and Guba (40) (Table 1).

**Table 1:** Four-dimension criteria (FDC) of qualitative research quality assurance.

Rigorous criteria	Purpose	Strategy used in this study
Credibility (Internal validity)	To guarantee authentic and credible results (from respondents’ perspectives)	• The interview length to 60–90 min for each interviewee. • Triangulation of data sources by age, sex and social status was made. • Preliminary finding was presented and discussed with respondents (member check) • Pilot test of KII guidelines before the interview process • MPH (master's degree holders) facilitate the interviews • All field notes and record were submitted for analysis
Transferability (External validity)	It is about the applicability or the degree to which the result of the study is transferred or generalized to another context	• Purposive intensity sampling • Probing of interview questions • Thick description of the data undertaken
Dependability (Reliability)	It is about consistency or reproducibility of the finding within the same group	• Data collection protocol was strictly followed • For audit trail detailed track records was taken
Conformity (Objectivity)	To boost confidence that the result would be verified by other researchers	• Analysis of results were also cross checked by other investigators

### 2.7 Data processing and analysis

The collected data were entered into Epi-data version 3.1 and exported to SPSS version 25 for analysis. Descriptive data were presented in the form of tables, graphs, text, and percentages. Binary logistic regression was used to establish the presence of an association between independent and dependent variables. During bivariate analysis, thirteen variables with *P*-values less than 0.25 were put into the multivariate logistic regression model. The model met all required assumptions, such as having a variance inflation factor (VIF) < 5, and its goodness of fit was assessed using the Hosmer-Lemeshow test. Then, using odds ratios and 95% confidence intervals, associations and their statistical significance were declared. A confidence interval and *P*-value were employed in the final model to claim statistical significance. For qualitative research, interviews were verbatim transcribed and translated into English. The English translations and the Afan Oromo transcriptions were compared. The data were then quoted, categorized, and thematized to generate thematic patterns. Finally, qualitative evidence was used to supplement the quantitative findings.

### 2.8 Ethical clearance

Ethical approval was provided by the Salale University (SLU) Institutional Review Board (IRB) with Ref. No. SLU-IRB/04/23. To communicate with the respective administrative bodies in the study area, a letter of cooperation was obtained from the Dire district administration office. After receiving permission from the administrative body, the aim and purpose of the study, including procedures, potential risks, and benefits, were explained to each study participant. The information's confidentiality was protected in such a way that no specific person could be identified. Respondents were assured that they had the right to refuse participation or terminate their participation at any time. Finally, each individual was asked to give their written consent.

## 3 Result

### 3.1 Socio-demographic characteristics of the participants

A total of 298 women were interviewed for this study, yielding a 97.7% response rate. Participants in the study had a median age of 32 years (IQR +10). Nearly three-quarters of participants, 208 (69.8%), as well as their partners, 181 (60.7%), lacked a formal education. Two hundred participants (67.8%) were housewives, with 157 (52.7%) of their husbands being farmers. In terms of household income, 119 (39.9%), 120 (40.3%), and 59 (19.8%) were classified as poor, medium, and rich, respectively. Around half of the participants, 141 (47.3%), possessed decision-making autonomy regarding family health, and 194 (65.1%) had no caretaker at home while waiting at maternity waiting home. A total of 217 (72.8%) use motor vehicles to go to the health facility, of which 168 (56.4%) do so in less than or equal to 60 min by foot (Table 2).

**Table 2:** Socio-demographic characteristics among pastoralist mothers in Dire district, Ethiopia, 2023 (*n* = 298).

Socio-demographic variables	Category	Number	%
Age	Less than or equal to 20	6	2
	21–25	46	15.4
	26–30	75	25.2
	31–35	76	25.5
	Greater than or equal to 36	95	31.9
Marital status	Married	260	87.2
	Divorced	28	9.4
	Widowed	10	3.4
Ethnicity	Oromo	265	88.9
	Amhara	8	2.7
	Burji	12	4
	Others^a^	13	4.4
Religion	Orthodox	25	8.4
	Protestant	109	36.6
	Muslim	118	39.6
	Wakefata	46	15.4
Educational status of participants	No formal education	208	69.8
	Primary education	68	22.8
	Secondary and above	22	7.4
Educational status of partners	No formal education	181	60.7
	Primary education	49	16.4
	Secondary and above	68	22.8
Household wealth	Poor	119	39.9
	Medium	120	40.3
	Rich	59	19.8
Occupation of participants	Workers^b^	96	32.2
	House wife	202	67.8
Occupation of partners	Non-farmers^c^	141	47.3
	Farmers	157	52.7
Family Size	Less than 5	104	34.9
	Greater than or equal to 5	194	65.1
Decision-making autonomy of participants	Yes	141	47.3
	No	157	52.7
Decision maker for un-autonomous (*n* = 157)	Husband/partner	64	40.8
	Jointly	93	59.2
Partners support to wait at maternity waiting home	Yes	254	85.2
	No	44	14.8
Mother/father-in-law support to wait at maternity waiting home	Yes	255	85.6
	No	43	14.4
Neighbors support to wait at maternity waiting home	Yes	296	99.3
	No	2	0.7
Having children at home who need care	Yes	257	86.2
	No	41	13.8
Having a caretaker at housework	Yes	104	34.9
	No	194	65.1
Possibility of getting an attendant to wait at maternity waiting home	Yes	158	53.0
	No	140	47
Acceptable maternity waiting home time 2–4 weeks	Yes	76	25.5
	No	222	74.5
Distance to the health facility in minutes	≤60	168	56.4
	>60	130	43.6
Means of transportation to access health institution	Vehicles	217	72.8
	By foot/walking	81	27.2

^a^ Silte, Somali, Wolaita, Gedeo.

^b^ Farmers, Merchant, Government employee, Daily laborer.

^c^ Merchant, Government employee, Daily laborer.

### 3.2 Obstetrics and reproductive characteristics of participants

The majority, 238 (80%), of the mothers became pregnant when they were older than 20 years. Two hundred eighty-three (95%) of the Participants were received ANC follow-up during pregnancy. Besides, two hundred thirty-two (77.9%) of the participants had received maternity waiting home information and counseling. The majority of participants, 254 (85.2%), had two or more children, whereas 86.6% of them had a child before their most recent delivery at a health facility (Table 3).

**Table 3:** Obstetrics/reproductive characteristics among pastoralist mothers in Dire district, Ethiopia, 2023 (*n* = 298).

Obstetrics/reproductive variables	Category	Number	%
Age at first pregnancy	Less than or equal to 20	60	20.1
	Greater than 20	238	79.9
ANC follow up during current pregnancy	Yes	283	95
	No	15	5
Frequency of visit	One or no visit	35	11.7
	Two or more visit	263	88.3
Gestational age in weeks at 1st visit	Less than or equal to 14	8	2.7
	15–28	255	85.6
	≥29	35	11.7
Number of deliveries	Once	44	14.8
	Two & above	254	85.2
A place of birth of child preceding the youngest infant (*n* = 254)	Health facility	220	86.6
	Home	34	13.4
Difficulty faced during preceding pregnancy and delivery (*n* = 254)	Yes	80	31.5
	No	174	68.5
Past experience of maternity waiting home use	No past experience of use	292	98
	Has past experience of use	6	2

### 3.3 Knowledge of the danger signs of pregnancy

Two hundred forty-three (83.2%) were aware of the potential danger signs of pregnancy, which included convulsions, fever and headache with impaired vision, and vaginal bleeding, with response rates of 222 (31.7%), 143 (20.4%), and 128 (18.3%), respectively. In general, 136 (45.6%) women had poor knowledge, whereas 162 (54.4%) had good knowledge (Table 4).

**Table 4:** Knowledge of mothers on danger sign of pregnancy, and attitude of mothers towards maternity waiting home & CRC of health professionals in Dire district, Ethiopia.

Knowledge and attitude variable	Category	Number	%
Information of maternity waiting home and its importance	Yes	225	75.5
	No	73	24.5
Counselled about maternity waiting home during ANC visit	Yes	232	82
	No	51	18
Importance of maternity waiting home for mothers’ responses’	Prevents death due to complication	124	37.9
	Mothers can get early postnatal	61	18.7
	Mother can get health information	32	9.8
	Deliver safely without fear	110	33.6
Role of maternity waiting home on child health responses (*Multiple responses are possible*)	Prevents death due to complication	87	33
	Newborn can get early postnatal	50	18.9
	Newborn can get immunization	127	48.1
Danger sign of pregnancy responses’ (*Multiple responses are possible*)	Vaginal bleeding	128	18.3
	Convulsions/fits	222	31.7
	Fever, headaches with blurred vision	143	20.4
	Fever and too weak to get out of bed	44	6.3
	Severe abdominal pain	112	16.0
	Fast or difficult breathing	52	7.4
Do you know danger sign of pregnancy	Yes	248	83.2
	No	50	16.8
Knowledge of danger sign of pregnancy	Poor knowledge	136	45.6
	Good knowledge	162	54.4
Attitude towards maternity waiting home use (*n* = 298)	More negative attitude	66	22.1
	More positive attitude	232	77.9
Attitude towards CRC of health workers (*n* = 105)	More negative attitude	20	19
	More positive attitude	85	81

### 3.4 Attitude towards maternity waiting home and CRC of health professionals

Sixty-six (22.1%) women had a more negative view regarding maternity waiting homes, whereas two hundred thirty-two (77.9%) had a more favorable perspective. Similarly, 85 (81%) had a more favorable view regarding the CRC of health professionals (Table 4).

### 3.5 Maternity waiting home use

**Figure 3:**
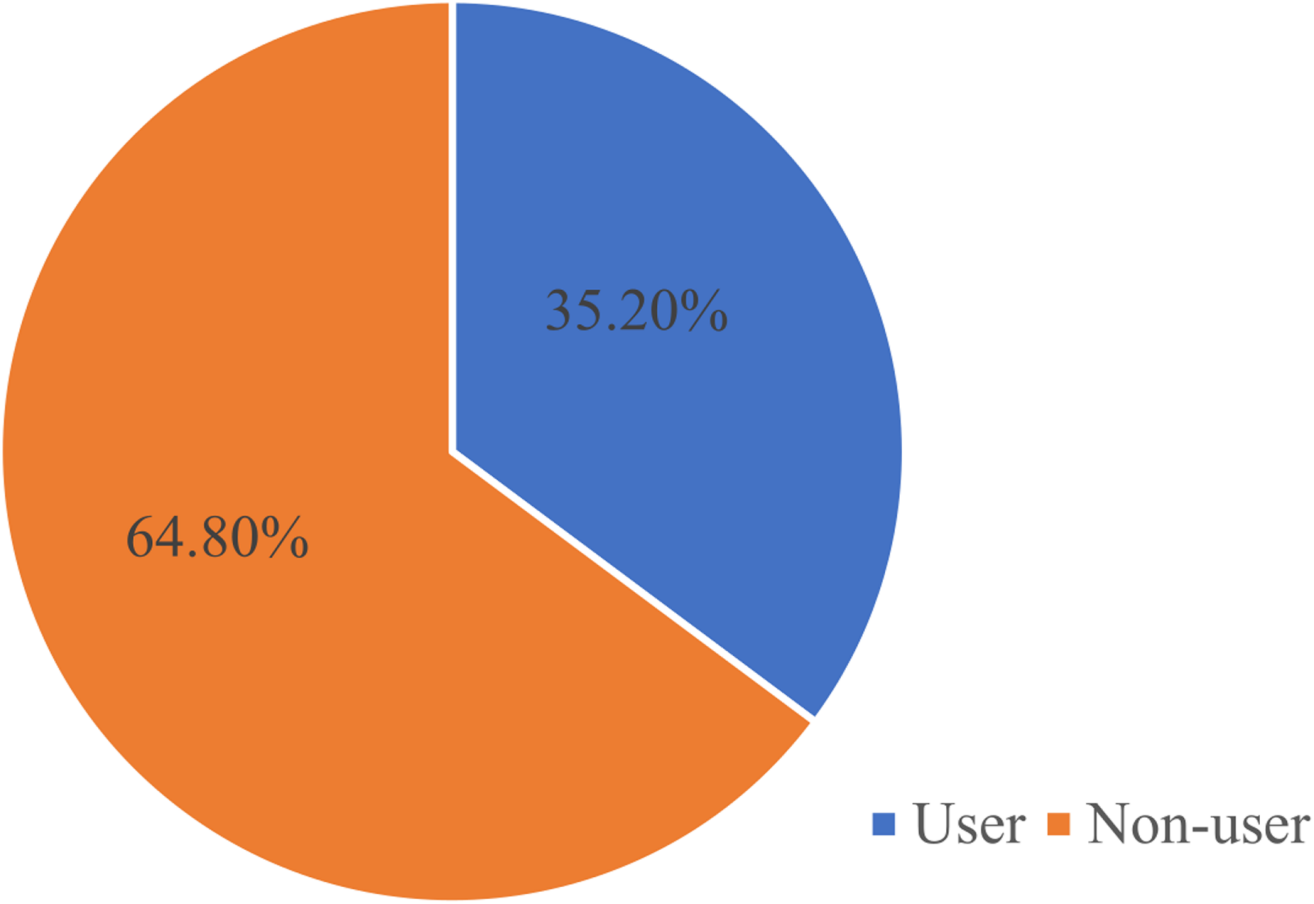
Prevalence of maternity waiting home use among pastoralist mothers in Dire district, Ethiopia, 2023 (*n* = 298).

In this study, the prevalence of maternity waiting home utilization was 105 (35.2%) (95% CI: 30.9%, 39.5%) (Figure 3).

### 3.6 Reason for non-use of maternity waiting home

**Figure 4:**
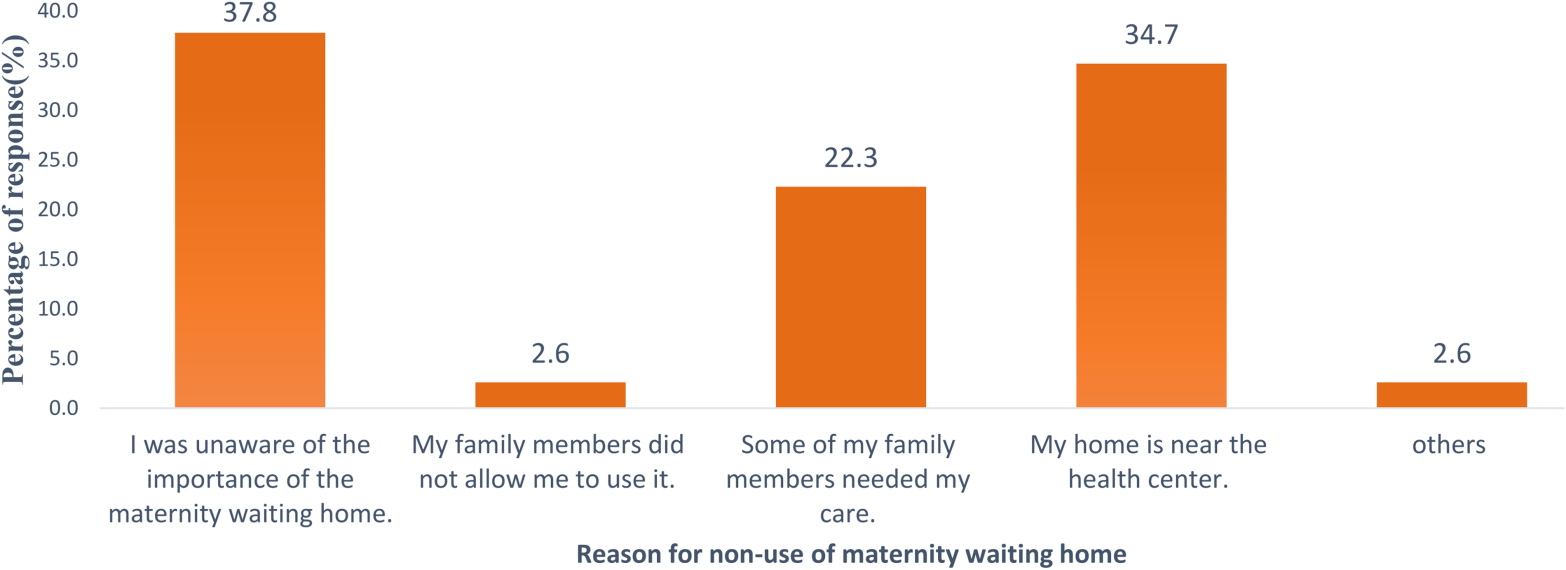
Reasons for non-use of maternity waiting homes among pastoralist mothers in Dire district, Ethiopia, 2023 (*n* = 298).

The major reasons for non-use of maternity waiting home were a lack of awareness about the existence of a maternity waiting home service 73 (37.8%), the proximity of the health facility to their living place 67 (34.7%), and the presence of family members who seek their care 43 (22.3%) (Figure 4).

### 3.7 Facility-related services

About ninety-three (88.6%), seventy-four (70.5%), and seventy-three (69.5%) of participants mentioned a lack of sleeping rooms for attendants, the traditional coffee ceremony, and cultural meals at maternity waiting home, respectively. Alongside this, one hundred (95.2%) women reported the possibility of ritual practice at maternity waiting home after childbirth. Moreover, ninety-six (91.4%) were pleased, while nine (8.6%) were unsatisfied with the service provided by maternity waiting home. Furthermore, the most frequently mentioned reason for satisfaction was the presence of bed 73 (28.5%) and food 72 (28.1%), whereas being away from family 7 (77.8%) was a repeatedly reported reason for not being satisfied (Table 5).

**Table 5:** Facility related services status among maternity waiting home users in dire district, Ethiopia, 2023 (*n* = 105).

Facility related variable	Category	Number	%
Availability of TV at maternity waiting home	Yes	57	54.3
	No	48	45.7
Availability of sleeping room for attendant	Yes	12	11.4
	No	93	88.6
Availability of privacy screen at maternity waiting home	Yes	81	77.1
	No	24	22.9
Possibility of bringing children to maternity waiting home	Yes	66	62.9
	No	39	37.1
Availability of cultural coffee ceremony during stay	Yes	31	29.5
	No	74	70.5
Availability of cultural food according to their needs	Yes	32	30.5
	No	73	69.5
Possibility of ritual practice at maternity waiting home after child birth	Yes	100	95.2
	No	5	4.8
Have you satisfied during the stay	Yes	96	91.4
	No	9	8.6
Reason for satisfaction	Presence of cooker	23	9
	Presence of beds	73	28.5
	Presence of food	72	28.1
	Service provision	68	26.6
	Presence of space	12	4.7
	Others^a^	8	3.1
	Total	256	100
Reason for not satisfied	Poor service provision of health professions	2	22.2
	I was departed from family	7	77.8
	Total	9	100

^a^ Others: Presence of toilet and water.

### 3.8 Factors associated with maternity waiting home

Bivariate analysis was used to identify prospective candidate variables for final multivariate analysis, and variables with *P*-values less than 0.25 were chosen as candidate variables. Accordingly, participants’ education, partners’ education and occupation, family size, decision-maker on family health, gestational age at the first visit, partners’ support, the possibility of getting attendants to wait at maternity waiting home, place of birth of preceding delivery, the difficulty faced during preceding pregnancy and delivery, travel time, the acceptable waiting time of 2–4 weeks, having caretaker for household tasks, and knowledge on danger signs of pregnancy were candidates for multivariate logistic regression. Then, to discover independent determinants of maternity waiting home consumption, a multivariate logistic regression model was fitted. Finally, after controlling for other potentially confounding variables, travel time of more than 60 min from a nearby maternity waiting home, good knowledge of danger signs of pregnancy, lack of caregivers for household tasks, and refusal to accept waiting time of 2–4 weeks were associated with maternity waiting home utilization.

Accordingly, a woman who travels more than 60 min on foot to reach a local maternity waiting home facility is five times more likely to use it than a woman who travels less than 60 min on foot (AOR 95% CI: 5.47, 1.77, 16.91). A woman with adequate awareness of pregnancy risk signs was also five times more likely to use maternity waiting home than their counterpart (AOR 95% CI: 5.41, 1.86, 15.79). A woman who had no other family to help with housework was 90% less likely to use the maternity waiting home than a woman who had other families to help with housework (AOR 95% CI: 0.10, 0.03, 0.31). When compared to women who had accepted a waiting time of 2–4 weeks, women who had refused to accept a waiting time of 2–4 weeks were 77% less likely to use maternity waiting home (AOR 95% CI: 0.24, 0.06, 0.74) (Table 6).

**Table 6:** Factors associated with maternity waiting homes utilization among of pastoralist mothers in Dire district, Ethiopia, 2023 (*n* = 298).

Associated factors	Category	Maternity waiting home use		COR^a^	AOR^b^ (95% CI)	*P*-value
		User	Non-user			
		*n* (%)	*n* (%)			
Travel time	≤60 min	28 (26.7)	140 (72.5)	1	1^c^	
	>60 min	77 (73.3)	53 (27.5)	7.26 (4.23,12.41)	5.47 (1.77, 16.91)	0.003
Knowledge on danger sign of pregnancy	Poor knowledge	36 (34.3)	100 (51.8)	1	1	
	Good knowledge	69 (65.7)	93 (48.2)	2.06 (1.26,3.37)	5.41 (1.86, 15.79)	0.002
Decision maker on family health	Husband/partner	10 (24.4)	54 (46.6)	1	1	
	Jointly	31 (75.6)	62 (53.4)	0.43 (0.26, 0.69)	0.68 (0.19, 2.40)	0.55
Place of birth of child preceding young infant	Health facility	70 (66.7)	150 (77.7)	1	1	
	Home	22 (21)	12 (6.2)	3.93 (1.84, 8.39)	0.14 (0.01, 1.75)	0.128
Having care-taker at house work	Yes	64 (61.0)	40 (20.7)	1	1	
	No	41 (39.0)	153 (79.3)	0.17 (0.1, 0.28)	0.10 (0.03, 0.31)	0.000
Family size	<5	31 (29.5)	73 (37.8)	1	1	
	≥5	74 (70.5)	120 (62.2)	1.45 (0.87,2.42)	0.77 (0.24,2.49)	0.667
Acceptable waiting time 2–4 weeks	Yes	39 (37.1)	37 (19.2)	1	1	
	No	66 (62.9)	156 (80.8)	0.4(0.24, 0.68)	0.24(0.08, 0.74)	0.014

^a^ COR, crude odds ratio.

^b^ AOR, adjusted odds ratio.

^c^ 1, reference.

### 3.9 Model of goodness of fit test

The Hosmer-Lemeshow test, with a *P*-value of 0.744, suggests that the logistic regression model demonstrates a satisfactory fit to the data. There is a lack of robust evidence indicating systematic misfit. The Cox & Snell R Square (0.311) and Nagelkerke R Square (0.45) metrics, which quantify the proportion of variance elucidated by the model, signify an improved fit and imply that the model accounts for a segment of the variability in the outcome. Hence, based on the evidence, these variables within the equation can be deemed reliable predictors of maternity waiting home usage among women who gave birth within the last year in the Dire district, southern Ethiopia.

### 3.10 Results of the qualitative study

Three community representatives, as well as three personnel from the district health office, were included in the KII to add to the quantitative study. The finding highlights three key themes and ten categories affecting the utilization of maternity waiting homes. The first theme discusses resource shortages and management issues, including problems with food supply, community contributions, and food preferences and services mismatch. The second theme reveals challenges in maternity care, such as extended stays, a lack of regular health checkups, and a skill gap among health workers. In addition, a shift in focus from maternity waiting homes due to improved skilled birth attendance and ineffective community bylaws is noted. The third theme explores the lack of awareness and lifesaving potential of maternity waiting home where there is limited network access. Here are the descriptions:

#### 3.10.1 Theme one: resource shortages and management issues

##### 3.10.1.1 Insufficient food

Despite the reassurances given by HEWs regarding the well-equipped facilities of maternity waiting homes and the promise of free accommodation until childbirth, troubling concerns arose about the insufficient food supplies and the associated expenses. A respondent from the community, a 42-year-old female, touchingly expressed her disappointment*:*

“ …… We were assured by HEW that maternity waiting home is adequately equipped and that women can reside until childbirth at no cost. However, users are distressed by the lack of food and the financial burden it entails …. ”

##### 3.10.1.2 Community involvement and resource use

The interview provided an in-depth look at the community's active support for maternity waiting home services, emphasizing their financial contributions and cereal donations. It also discussed the challenge of ensuring resources reach beneficiaries in a timely manner and are used effectively. A 45-year-old male from the community remarked:

“ *…* Even though the community donates once a year both in cash and in kind, there is a food scarcity due to collection through use mishandling *…*. ”

In addition, 38-year-old female from a health office stressed:

“ …. While each household is expected to contribute annually to feed mothers, some households fail to do so in a timely or appropriate manner. “Besides, there are notable delays in transferring the accumulated resources to the service site …. ”

Furthermore, the prolonged drought in the area has exacerbated the already alarming cereal shortages experienced by mothers. A 31-year-old male from a health office elucidated:

“ … The prolonged and severe drought has led to a significant shortage of food supplies for households, prompting communities to cease their contributions towards feeding mothers. Consequently, fewer mothers are able to access maternity waiting homes through healthcare financing …. ”

##### 3.10.1.3 Food preferences and services mismatch

Alongside food shortages, the effectiveness of services is further compromised by a gap between what pregnant women prefer to eat and the food provided at maternity waiting home. Supported by a quote from a 43-year-old female from the community:

“ …. During pregnancy, women typically consumed milk and dairy products at home, but they don't have access to these at maternity waiting home, where rice and tea are the primary offerings …. ”

It was further strengthened by the quotes from a 42-year-old female from the community:

“Women need masala tea (sweetened tea with milk) during breakfast; however, maternity waiting home provides black tea instead”.

A 33-year-old man from a health office highlighted the absence of a daily coffee ceremony for awaiting mothers at the residence

“ …… Although there isn't a daily coffee ceremony, a cultural coffee in the community to mark childbirth was observed at maternity waiting home …. ”

#### 3.10.2 Theme 2: challenges in maternity care and changing priorities

##### 3.10.2.1 Extended stay at maternity waiting home

The considerable responsibilities women bear in managing household duties were found to be challenging during extended stays at maternity waiting homes. Here is a quote from a 43-year-old female from the community to support this statement:

“ …. Prolonged stays at maternity waiting home are emotionally challenging for women who have left their children and domestic responsibilities behind …. ”

##### 3.10.2.2 Lack of regular health checkups

The lack of regular checkups and limited interaction with healthcare providers has resulted in dissatisfaction and a pessimistic view of the homes, potentially acting as barriers to utilizing the services. Here it is illustrated by a 42-year-old female from the community:

“Despite maintaining regular communication with the cooker, many express dissatisfaction and disappointment due to the inconsistent checkups and visits by health professionals, which they eagerly anticipate”.

##### 3.10.2.3 Skill gap in maternity care

Despite the lack of regular checkups, turnover among trained health workers was a challenge encountered:

“ … High turnover of trained and experienced health workers leads to a skill gap in offering good maternity service …. ” (38-year-old female from a health office).

In addition, a skill gap among HEW was highlighted here:

“ … HEW often fail to estimate the pregnancy ages of mothers who are eligible to wait at maternity waiting home …” (a 43-year-old female from the community).

##### 3.10.2.4 Focus shift

Efforts in advocacy and promotion have notably increased the utilization of maternity waiting homes. However, substantial progress in achieving the intended indicator has led to a shift in focus towards alternative priorities. This is supported by a quote from a 31-year-old male from a health office:

“ …. Following the improvement of SBA in our district, our focus shifted from maternity waiting home, as its purpose was to facilitate institutional delivery … ”

##### 3.10.2.5 Ineffective community bylaw

In addition to continuous education and promotion efforts on maternity waiting homes in the community, it is advisable to enact and enforce a by-law prohibiting home deliveries. This is because, when effectively implemented, women in rural areas have regularly chosen to stay in these residences to avoid financial penalties. Here it is backed by a quote from 42-year-old woman from the community.

“ ….. A rigorous community bylaw that fined home-delivered women with a 500-birr fee was no longer in effect, so women living far away are delivering at home, and use is declining …… ”

#### 3.10.3 Theme 3: lack of awareness and limited network connectivity

##### 3.10.3.1 Lack of community awareness

Furthermore, limited interaction with healthcare facilities and residing at a distance can affect their level of comprehensive understanding of these facilities.

“ … Despite the limited awareness of the availability of maternity waiting homes at health facilities, many community members residing far away and having limited contact with health facilities lack a profound understanding of the purpose of these homes …. ” (a 45-year-old male from the community).

Despite this, there is a prevalent belief within the community that these services are exclusively for mothers facing complications, leading to the misconception that the stay of healthy mothers at maternity waiting home is a waste of time.

“ …. A healthy mother, without complications and with ease of access to healthcare facilities during labor, is mistakenly residing at the maternity waiting home, leaving her family behind …. ” (42-year-old female from the community).

##### 3.10.3.2 Impact of accessibility and connectivity

Lack of network connectivity in certain areas has tragic consequences during labor, emphasizing the life-saving potential of maternity waiting homes. The absence of a network can hinder timely communication with ambulances, potentially putting women's lives at risk.

“ …. In certain villages, there is no network connectivity nearby to contact an ambulance during labour; I observed a woman die on the route to the hospital; had she stayed at maternity waiting home, her life would have been saved … ” (45-year-old male from the community).

## 4 Discussion

The finding of this study revealed that the prevalence of maternal waiting home utilization among women who gave birth in the previous 12 months in the study area was 35.2% (95% CI: 30.9%, 39.5%). This finding is lower than the study in the Sidama zone (20) and Gimbo district (41), where 67.25% and 42.5% of women had used maternity waiting home services, respectively. This might be due to maternity waiting homes being established in these areas a long time ago, and the participant's awareness about the service might increase or regional differences in implementing nationally suggested maternal healthcare policies. Nonetheless, the findings were similar to a study conducted in the Finfinnee special zone, where 34% of women used maternity waiting home (23).

However, it was greater than the finding of the study in the Digalu and Tijo district (33), Dabat district (11), East Bellessa district (21), and Jinka hospital (19) in which about 23.6%, 16.2%, 20.5%, and 16.7% of the study participants were reported to use maternity waiting home service, respectively. The disparity might be related to a difference in the study environment in which this study was conducted in transformed district where there is support from several stakeholders, notably the “Cordaid” performance-based financing (PBF) approach, which began in the area as pilot project in 2015. Beside this, higher exposure to maternity waiting home information during ANC visits, as well as the highest ANC coverage in the study location, may contribute to variance, as research has shown a substantial association between maternity waiting home consumption and ANC (31, 33).

Furthermore, it aligned with results from a study in Tanzania, where 31.3% of women used maternity waiting homes (42). However, this figure exceeded the utilization rate observed in Rwanda, where 25.2% of women had accessed maternity waiting home (7). This could be attributed to differences in the socio-cultural characteristics of the research population, as well as numerous initiatives and strategies implemented by the Ethiopian government, such as recruiting HEW at the community level, establishing and working with the Women Development Army (WDA), and promoting and expanding community-financed maternity waiting home at health centers.

This study also found that women who travel more than 60 min on foot to reach a local maternity waiting home facility are 5.47 times more likely to use the maternity waiting home compared to those who travel less than 60 min. This may be related to a fear of developing complications during delivery as well as the difficulty of obtaining transportation for necessities. The finding was consistent with research in the Digalu and Tijo district, the rural area of the Finfinnee special zone and Jimma (22, 23, 30). Besides, it is also in line with study findings in Tanzania and Indonesia (16, 42).

A woman with a good understanding of risk signs of pregnancy was 5.4 times more likely to use maternity waiting home than a woman with poor understanding of danger signs of pregnancy. One plausible reason is that persons who are aware of potentially life-threatening illnesses are more cautious and worried about their health than their counterpart. The findings were similar to research conducted in the Dabat district (11) and East Bellesa district (31). The key informant interviews also revealed that a lack of understanding regarding the importance of maternity waiting home poses a significant obstacle to its use. In addition, there is a widespread misconception at the community level that a mother residing at maternity waiting home has experienced pregnancy complications and is unable to give birth at home.

It is also in line with qualitative finding in southwest Ethiopia that elicit lack of understanding of consequence following home delivery as barrier of maternity waiting home utilization (37).

Moreover, women who lack caregivers for household tasks were 90% less likely to use the maternity waiting home compared to those who have support. This might be because women are the primary caregivers in the home, and until they delegate their responsibilities to others, it will be difficult for them to leave their family and stay in the maternity waiting home. The finding was similar to previous research in Dabat district, Gimbo district, and Indonesia (11, 41, 43). In addition, a qualitative study at Attat hospital and in south-west Ethiopia give high emphasize to the role of family support for home care during maternity waiting home utilization (37, 44).

Furthermore, compared to women who accept a 2–4-week stay, women who had refused to accept were 77% less likely to use maternity waiting home. This might be because women prefer to return to house work after a short stay at maternity waiting home. This finding is consistent with study in the Dabat district (11). Likewise, a key informant mentioned that extended stays at maternity waiting home can be emotionally difficult for women who have left their children and household responsibilities behind, as they are the primary caregivers.

A qualitative finding from an Ethiopian rural health facility also supports this, exploring longer stays in maternity waiting homes as an impediment women encountered at maternity waiting home (15).

### 4.1 Strength

This study was based in a pastoralist area of the Oromia regional state, which was not more focused by previous studies. In addition, it was done at the community level with mixed methods of data collection to get the full picture. Moreover, actual utilization was measured directly by interviewing delivered women, as opposed to the majority of previous studies, which measured the intention to use a maternity waiting home. Furthermore, pre-tests prior to data collection and the reliability of the tools (Cronbach's alpha > 0.85) also enhance the validity of this study.

### 4.2 Limitation of the study

Because of the potential for forgetting events that occurred one year ago, this study could not avoid recall bias. However, this bias is likely modest as childbirth is a memorable event. Social desirability bias might also be present, as the data collectors were community-level workers familiar with the participants, which may have influenced the participants to withhold their actual practices. To mitigates this bias, the data collectors were relocated to gather data outside of their usual work areas.

Moreover, the design effect was not accounted for due to time and resource limitations. This oversight may have led to an underestimation of standard errors and an increase in Type I error rates. Neglecting the design effect could also have resulted in overstating the significance of our findings, thus raising the risk of false positive results.

## 5 Conclusion

The prevalence of the maternity waiting home's utilization in the study area was low when compared to the national interest to promote this service by the pregnant mothers. Longer travel times, good knowledge of pregnancy danger sign, refusal to accept waiting times of 2–4 weeks and lack of a caregiver at home were among the identified predictors of maternity waiting home utilization in the study area.

Thus, active community involvement, both financially and in other forms, is crucial for sustaining maternity waiting home services. Dire district health office is recommended to enhance coordination with the WDA and other social networks to address caregiver-related issues and social challenges. Effective health education and counseling by health extension workers and other health care providers on pregnancy risk signs are also important during ANC visits and in grassroots communities. Moreover, service providers in collaboration with policy makers should work to reduce long waiting times, considering the significant household responsibilities that women often have. Furthermore, a collaborative effort between district health offices and local NGOs is necessary to fill the skill gap in the workforce. Household's members would need to cover the responsibility of pregnant mothers as care providers to ensure the effective use of maternity waiting homes for pregnant mothers.

## Data Availability

The original contributions presented in the study are included in the article/Supplementary Material, further inquiries can be directed to the corresponding author.
