# Supplementary material for: Utilization and determinants of maternity waiting homes among pastoralist mothers in Dire district, southern Ethiopia: a mixed-methods study
Source: Front Glob Womens Health. 2024 Oct 25;5:1446500. doi: 10.3389/fgwh.2024.1446500 (PMC11543576; doi:10.3389/fgwh.2024.1446500)
Supplement: Supplementary file 1 [file Datasheet1.pdf]

ANNEX I: INFORMATION SHEET AND CONSENT FORM:

Information sheet

Hello! My name is. ______________________

I'm here to gather data for the MPH in General Public Health thesis that Hassan Mahamad, the principal investigator, from Salale University's College of Health Science, is working on. This study will be carried out in the Dire district, Borana zone on women who gave birth within the previous year, and you were selected as a participant, and before getting your consent, you need to know all necessary information related to the study, which was detailed as follows:

Introduction

In the context of health care, privacy refers to the duty of a care provider to protect a client from any disclosure (i.e., discovery by others) of personal health data by ensuring protection to the patient and the patient's records. Privacy is the state of not being subject to intrusion. Contrarily, confidentiality is the restriction of information to those who need it. As a result, this information sheet briefly outlines the essential considerations for the investigation.

Objective: To assess maternity waiting home utilization and associated factors among women who gave birth within the previous one year in the Dire district, Borana Zone, June 2023. Participants to be included: All women who gave birth in Dire district in the last year and have lived there for at least six months

Risks and discomfort: There are no risk in participating in this study. However, there might be discomfort in answering questions, which will last up to 30 minutes.

Benefits: There is no immediate benefit for participating in this study. However, your participation will help to improve the health-care delivery systems services in the future.

Incentive: There is no participation fee or material incentive for participating in this study.

Confidentiality: The information that we will collect for this study will kept confidential. Information about you that collect for the study will stored in a file, which will not have your name on it but a code number assigned to it. It will not be revealed to anyone except the investigator.

Participant Rights Your participation is up to you to decide. You have the right not to participate or answer any questions you do not want. When you need to interrupt, you can do so at any time. However, your truthful response will determine how well this study turns out.

Persons to contact: If you have any questions, you can ask them at any time. If you have additional questions about the study, you can contact the investigator:

Mr. Hassan Mahamad

Phone numbers +251926503490 Thank you for your cooperation!!

If you are volunteer to participate, we kindly request that you provide your response

Consent form

In understanding this document, I am giving my consent to participate in the study that was carried out in the Dire district, Borana Zone on women who gave birth within the previous year. I have been informed of the purpose of this study. I have understood that participation in this study is entirely volunteer. I have been told that my answers to the questions will not be given to anyone else, and no reports of this study will ever identify me in any way. I have also been informed that my participation or non-participation or my refusal to answer questions will have no effect on me. I understood that participation in this study does not involve any risks. I understood that Hassan Mahamad is the person to contact if I have questions about the study or about my rights as a study participant.

Respondent's Name____________________Signature_______________Date__________

Interviewer Name _________________________Signature ___________ Date__________

ANNEX II-ENGLISH VERSION QUESTIONNAIRE

Questionnaire identification code

001.Questionnaire identification code: _______002.Data collectors' code: ________

003.Supervisor's code: ______ 004.Date of data collection:______day\month

Instruction:

It was prepared and given to trainees (supervisors and data collectors) to use at the time of data collection.

Skip the irrelevant questions and move on to the ones that are relevant.

Instead of mentioning options as an alternative or choice for participants, encourage them to mention them themselves (via probing).

Check the collected data on a daily basis to identify trends and take corrective action in a timely manner.

Incomplete data needs to be completed the next day to ensure its completeness prior to starting the daily activity.

Anything that occurs must be clearly reported to the supervisor or principal investigator.

Part I: Socio-Demographic Characteristics

Field Interviewer name: ________________Interview date ______participant's Code___

No	Variables	Categories	Skip
101	How old are you?	( ) years	
102	What is your marital status?	1.Married 2. Single 3. Divorced 4. Widowed	
103	Ethnicity	1.Oromo 2. Amhara 3. Tigre 4. Burji 5. others	
104	Which religion do you practice?	1.Orthodox 2. Protestant 3. Muslim 4. Catholic<br>5. Wakefata	
105	What is the highest level of education you have attended?	1.Illiterate 2. Read and write 3. Elementary school (grade 1 -4) 4. Secondary school (Grade 5-8)<br>5. High school/prep. (grade 9 -12) 6. Above grade 12	
106	What is your occupation?	1.Merchant 2. Farmers 3. Housewife<br>4.Government employee 5. Laborer 9. Other specify	
107	What is your husband's highest level of education?	1.Illiterate 2. Read and write 3. Elementary school (grade 1 -4) 4. Secondary school (Grade 5-8)<br>5. High school/prep. (grade 9 -12) 6. Above grade 12	
108	What is your husband's occupation	1.Farmers 2. Merchant 3. Government employee<br>4. Daily laborer 5. Other specify	
109	Family size	(___)	
110	Which of these are exist in your house?		
	Farmland	1. Yes 2. No	
	Cow	1. Yes 2. No	
	Ox	1. Yes 2. No	
	Horse	1. Yes 2. No	
	Donkey	1. Yes 2. No	
	Goat	1. Yes 2. No	
	Sheep	1. Yes 2. No	
	Chicken	1. Yes 2. No	
	Has electricity	1. Yes 2. No	
	Solar light	1. Yes 2. No	
	Kerosene lamp	1. Yes 2. No	
	Has refrigerator	1. Yes 2. No	
	Pipe Water	1. Yes 2. No	
	Improved Latrine	1. Yes 2. No	

	Has car	1. Yes 2. No	
	Bajaj	1. Yes 2. No	
	Has motorcycle	1. Yes 2. No	
	Has television	1. Yes 2. No	
	Has radio	1. Yes 2. No	
	Mobile phone	1. Yes 2. No	
	Chair	1. Yes 2. No	
	Table	1. Yes 2. No	
	Bed with Mattress	1. Yes 2. No	
	Health insurance	1. Yes 2. No	
	Others (specify)		
111	The main material of the roof (record your observation)		
	Hatch/leaf/ mud	1. Yes 2. No	
	Has corrugate iron roofing	1. Yes 2. No	
	Has roofing tiles	1. Yes 2. No	
	Has other roofing	1. Yes 2. No	
112	The main material of the walls (record observation)		
	No walls	1. Yes 2. No	
	Plywood	1. Yes 2. No	
	Cement	1. Yes 2. No	
	Rudimentary walls	1. Yes 2. No	
	Stone with cement	1. Yes 2. No	
	Stone with mud	1. Yes 2. No	
	Finished wall	1. Yes 2. No	
	Covered Adobe	1. Yes 2. No	

continue

continue

No	Variables	Categories	Skip
113	Can you decide alone on your health care?	1.Yes 2. No	If yes go to 115
114	If no to the above question: who decide for maternal and child health care needs?	1.Husband 2. Mother/father-in-law 3. Jointly 4. other specify	
115	Is your husband’s volunteer to wait in MWH?	1. Yes 2. No	
116	Are your mother/father-in-law volunteer to wait in MWH?	1.Yes 2. No	
117	Are your neighbors support you to wait in MWH?	1.Yes 2. No	
118	Do your culture support you to uses MWH?	1.Yes 2. No	
119	Do you have other children at home who need care?	1.Yes 2. No	
120	Can you bring attendant during waiting in MWH (is there permission)	1.Yes 2. No	
121	Do you have other family to do House hold responsibilities in your home?	1.Yes 2. No	
122	Can you get attendant during waiting time?	1.Yes 2. No	
123	Methods of travelling to access the health institution	1. Vehicle 2. Horses or any animals 3. Walking 4. Other	
124	How long did it take from your home to the place of delivery on foot?	____in minutes	
125	If you use vehicle: what is the traveling km from your house to MWH	_____ in Km	

Part II: Reproductive/obstetrics Characteristics

No	Variables	Categories	Skip
201	Age at first marriage	(--------)	
202	Age at first pregnancy	(--------)	
203	How many times have you gave birth?	1.Once 2. Two & More	If once skip to 208
204	If you have delivered two and above, how many years between the past and current infant (birth spacing)	(__) years	
205	If you delivered two and above, where have you delivered your first child?	1.Health facility 2. Home<br>3. Other specify	
206	If you delivered two and above have you faced any difficulty during pregnancy and delivery preceding the youngest infant?	1.Yes 2. No	
207	If your answer is yes: what type of difficulties do you encounter **(multiple response is possible)**	1.Ante-partum hemorrhage<br>2. Preterm labor<br>3.Delayed labor<br>4.post-partum hemorrhage<br>5.Neonatal asphyxia<br>6.Preeclampsia &eclampsia<br>7.Still birth 8. Other specify	
208	Have you received antenatal care for your most recent delivery?	1.Yes 2. No	If No go to301
209	If your answer is yes: when do you start? put your answer in weeks	(____)	
210	How many times did you receive Antenatal care during this pregnancy?	(____)	
211	During your ANC visits, did you receive information and counseling about MWHs	1.Yes 2. No	

Part III: Knowledge of mothers on MWH and its importance

No	variables	categories	Skip
301	Do you know about MWHs and its importance?	1.Yes 2. No	If no go to 303
302	If your answer to question number 301 is yes, mention importance you know (Multiple response is possible)	1.Prevents mother from death due to complication of pregnancy<br>2.Mothers can get early postnatal care.<br>3.Mother can get health information<br>4.Deliver safely without fear<br>5.Others specify…………	
303	Do you know danger signs of pregnancy?	1.Yes 2. No	If no go to 305
304	If your answer for question number 303 is yes, mention danger signs you know (Multiple response is possible)	1.Vaginal bleeding<br>2.Convulsions/fits<br>3.Fever headaches with blurred vision<br>4.Fever and too weak to get out of bed<br>5.Severe abdominal pain<br>6.Fast or difficult breathing.	
305	Do MWHs have roles on children health?	1.Yes 2. No	If no go to 307
306	If your answer for question number 305 is yes, mention roles you know (Multiple response is possible)	1.Prevents death due to complication<br>2.Newborn can get early postnatal care.<br>3.Newborn can get immunization<br>4.Others specify	
307	Do you know acceptable pregnant mothers admitting time/period to MWH?	1.Yes 2. No	If no go to 401
308	If your answer for question number 307 is yes, tell me the time/period	(____) in weeks	

Part IV: Attitude of mothers towards MWH utilization

No	**Attitude towards MWH utilization**	**Strongly agree=5**	**Agree=4**	**Neutral=3**	**Disagree=2**	**Strongly disagree=1**
401	MWHs are important for the health of mothers and infants					
402	It is worthwhile for pregnant to stay in MWH					
403	Going early to MWHs to wait for delivery is wiser than waiting at home until labor					
404	Waiting for delivery at MWHs prevents pregnant women from reaching the health facility late due to long distances and lack of transport					
405	Separation of pregnant women from husband and children to stay at MWH for delivery does not hurt them					
406	Waiting at the MWH will help women find assistance from the nurses and midwives if they develop labor complication					
407	A Facility with an MWH is more beneficial to the mother and baby than a facility without MWH					
408	Staying in the MWH while waiting for delivery will safely guard mothers.					

Part V: MWH utilization

No	variables	Categories	Skip
501	Did you stay at the maternity waiting home just prior to onset of labor during your most recent delivery?	1. Yes 2. No	If no skip to 506
502	If your answer for question number 501 is yes, what were your reasons? (Multiple answer is possible)	1.I had complication during my previous pregnancy<br>2.As my home is far from HC, it was to avoid delay related death.<br>3.Because I have benefited during my previous utilization<br>4.Health extension workers informed that my EDD had reached<br>5.Others, specify……………………	
503	If your answer to Q.501 is yes were MWHs fulfilled your interest?	1.Yes 2. No	If no skip to505
504	If your answer for question 503 is yes, what made you interested? (Multiple answer is possible)	1.Presence of cooker 2. Presence of beds 3. Presence of water 4. Presence of toilets 5. Presence of food 6. Presence of space 7. Service provision	
505	If your answer for question 503 is no, what made you disinterested? (Multiple answer is possible)	1.Health professionals were not providing service politely<br>2.Privacy was not kept<br>3. I was departed from family<br>4.Other, specify	
506	If your answer for question number 501is no, what were your reasons? (Multiple answer is possible)	1.I did not know about MWH and its importance<br>2. My family members did not allow me to use it<br>3.There were family members who seek my help<br>4. My home is near the health center<br>5.Others(specify)	

Part VI: facility related factors (Only for MWH user)

No	Variables	Categories	Skip
601	Is there kitchen in MWH?	1.Yes 2. No	If No go to 603
602	If answer to question No. 601 is yes, is there cooking and eating utensils?	1.Yes 2. No	
603	Is there functional own MWH latrine	1.Yes 2. No	
604	Is there sleeping room in MWH?	1.Yes 2. No	If No go to 607
605	If yes to question No 604, how many mothers sleep in one home?	(__)	
606	Is there sleeping room for person staying with the pregnant woman at the MWH	1. Yes 2. No	
607	Is there privacy screen in MWH?	1. Yes 2. No	
608	Is it possible to bring children to MWH?	1.Yes 2. No	
609	Can family visits you during maternity waiting home stay?	1.Yes 2. No	
610	Is there cultural coffee ceremony program?	1.Yes 2. No	
611	Is there cultural food which based on your culture or needs	1.Yes 2. No	
612	Is it possible to practice other local cultural practice in MWHs after child birth?	1. Yes 2. No	

Part VII: Attitude of mothers towards CRC of health professionals (only for MWH user)

No	Attitudes towards CRC	Strongly Agree=5	Agree=4	Neutral=3	Disagree=2	Strongly disagree=1
701	Health professions respects mothers in MWHs					
702	Health professions provide good care to mothers in MWHs					
703	Health professional working in MWH is compassionately serve the mothers					

Guide for key informants

Participant's information
Zone_______District_____Kebele_____Age_____Religious_____Educational background___
marital Status_____Occupation______Income Level______Date of interview_________
interviewer name___________start time__________End time_____

S.no	Guideline for key informants
1	What is maternal health problem in the area?
2	Where do women in this area give birth? Probe, Home, health facility and other
3	If home, why do you think they choice home?
4	Is the population of this area aware of the existence of maternity waiting home in the facility?
5	How could community obtain information regarding maternity waiting home?
6	Do woman utilizing MWH? what can you say about status of utilization?
7	Is there any community contribution to sustain MWH service?
8	in your own view, what do you think are the factors influencing the use of maternity waiting homes in this area? Probe Culture, service quality, distance…
9	What are the complaints of women you have heard on MWH use?
10	What do you think to be done to improve the utilization of MWHs in dire district? Probe, As government? Partners, community, health facility….
11	Do you have any general comment/idea you want to add

ANNEX III -AF-GAAFFILEE AFAAN OROMOO

Koodii garaa graa kan ragaaf kennamu

001.Koodii unkaa gaaffilee adda baasuu _______

002.Koodii funaantoota odeeffannoo: _________

003.Koodii supparvaayizaraa: _____

004.Guyyaa odeeffannoon itti walitti qabamu:____Guyyaa/ji'aa/Baraa

Qajeelfama

Qajeelfamni kun leenjifamtootaaf (supervaayizarootaa fi Daataa funaantootaaf) yeroo odeeffannoon walitti qabamutti akka fayyadamaniif kennama.

Gaaffilee barbaachisoo hin taane irraa garaa barbaachisoo ta'anitti darbi.

Filannoo hirmaattotaaf tarreessuu caalaa ofii isaanii akka kaasan jajjabeessi (karaa ittii argisiisi).

Daataa walitti qabame guyyaa guyyaan sakatta'uun adeemsa jiru adda baasuu fi tarkaanfii sirreeffamaa yeroon fudhachuu.

Daataan guutuu hin taane sochii guyyaa guyyaa jalqabuun dura guutuu ta'uu isaa mirkaneessuun guyyaa itti aanutti xumuramuu qaba.

Wanti uumamu kamiyyuu supparvaayizara ykn qorataa ijootti ifatti gabaafamuu qaba.

Kutaa I: Amaloota Hawaas-Dimoogiraafii

Maqaa ragaa funaanaa____________guyyaa ragaan funaaname____________Koodii Deebii kennitootaa_________

Lakk	Gaaffilee	Ramaddiiwwan	Irra kuti
101	Umuriin kee meeqa?	( ) waggaa	
102	Haalli gaa'ela keeti akkami?	1.Heerumeera 2. Hin heerumne<br>3.Hiikeera 4. Abbaan manaa lubbuun hin jiruu	
103	Sabni kee maalii	1.Oromo 2. Amhara 3. Tigre 4. Burji 5. Kan biroo	
104	Amantii kam hordoftu?	1.Ortodoksii 2. Pirootestaantii 3. Muslima<br>4.Kaatolikii 5.Waaqeeffataa	
105	Sadarkaan baruumsaa kee hammami?	1.Hin barannee 2. Dubbisuu fi barreessuu ni danda'aa 3. Barnoota bu'uraa (1-4)<br>4. Sadarkaa 2ffaa (5-8) 5. Kutaa 9-12 6. Kutaa 12 olii	
106	Hojiin keessan maali?	1.Daldala 2. Qonnaan bulaa 3. Haadha manaa<br>4.Hojjetaa mootummaa 5. Hojjetaa hojii humnaa<br>5. Kanneen biroo (ibsii)	
107	sadarkaan barumsaa abbaa manaa kee hammami?	1.Hin barannee 2. Dubbisuu fi barreessuu ni danda'aa 3. Barnoota bu'uraa (1-4)<br>4. Sadarkaa 2ffaa (5-8) 5. Kutaa 9-12<br>6. Kutaa 12 olii	
108	Hojiin abbaan manaa kee maali	1.Daldalaa 2. Qonnaan bulaa<br>3.Hojjetaa mootummaa 4. Hojjetaa hojii humnaa<br>5. Kan biroo (ibsii)	
109	Baay'ina maatii	(------) .	
110	Kanneen armaan gadii keessaa kami qabdu?		
	Lafaa qonnaa	1. Eeyyee 2. Lakkii	
	Loonii	1. Eeyyee 2. Lakkii	
	Qotiyyoo	1. Eeyyee 2. Lakkii	
	Fardaa	1. Eeyyee 2. Lakkii	
	Harree	1. Eeyyee 2. Lakkii	
	Re'ee	1. Eeyyee 2. Lakkii	
	Hoolaa	1. Eeyyee 2. Lakkii	
	Lukkuu	1. Eeyyee 2. Lakkii	

	Ibsaa	1. Eeyyee 2. Lakkii	
	Soolaraa	1. Eeyyee 2. Lakkii	
	Kurraazii	1. Eeyyee 2. Lakkii	
	Diilalleessa	1. Eeyyee 2. Lakkii	
	Saraaraa bishaanii	1. Eeyyee 2. Lakkii	
	Manaa fincaanii sadarkaa Eeggate	1. Eeyyee 2. Lakkii	
	konkolaataa	1. Eeyyee 2. Lakkii	
	Baajaajii	1. Eeyyee 2. Lakkii	
	motorsaayikilaa	1. Eeyyee 2. Lakkii	
	Televijiini	1. Eeyyee 2. Lakkii	
	Raadiyoo	1. Eeyyee 2. Lakkii	
	Bilbilaa Moobayilaa	1. Eeyyee 2. Lakkii	
	Barcumaa	1. Eeyyee 2. Lakkii	
	Minjaala	1. Eeyyee 2. Lakkii	
	Siree firaasha qabu	1. Eeyyee 2. Lakkii	
	Miseensaa inshuraansiiti?	1. Eeyyee 2. Lakkii	
	Kan biro (ibsii) _____		
111	Baaxiin manaa maali irraa hojjatame (waan ijaan argitee galmeessi)		
	Mukaa/Baala/dhoqqee	1. Eeyyee 2. Lakkii	
	Qorqoorro	1. Eeyyee 2. Lakkii	
	Bolokeetii	1. Eeyyee 2. Lakkii	
	Kan biroo	1. Eeyyee 2. Lakkii	
112	Girgiddaan manaa maal irraa hojjatame (waan ijaan argite galmeessi)		
	Girgiddaa maleessa	1. Eeyyee 2. Lakkii	
	Girgiddaa mukaa minjaalaatiin hojjatame (polywood wall)	1. Eeyyee 2. Lakkii	
	Simintoodhaan	1. Eeyyee 2. Lakkii	
	Girgiddaa bu'uraa(Rudimentary wall)	1. Eeyyee 2. Lakkii	
	Mukaa buqqa'ee hidhamu (reused wood)	1. Eeyyee 2. Lakkii	
	Dhakaa fi simintoon	1. Eeyyee 2. Lakkii	
	Dhakaa fi dhoqqeen	1. Eeyyee 2. Lakkii	
	Shaklaadhaan/fuulleen kan faayame	1. Eeyyee 2. Lakkii	
	Xuubii simintoo	1. Eeyyee 2. Lakkii	
	Xuubii dhoqqee	1. Eeyyee 2. Lakkii	

Ittii fufaa_____

Ittii fufaa…...

lakk	Gaaffilee	Ramaddiiwwan	Irra darbuu
113	Kunuunsa fayyaa kee irratti kophaa kee murteessuu dandeessaa?	1.Eeyyee 2. Lakki	Yoo eeyyee ta'e gara 115 deemi
114	Gaaffii armaan olii kanaaf lakki yoo ta’e: fedhii kunuunsa fayyaa haadholii fi daa’immanii eenyutu murteessa?	1.Abbaa manaa<br>2. Haadha/abbaa abbaa warraa<br>3. Waliin<br>4. Kanneen biroo	
115	Abbaan warraa kee iddoo turtii haadholii keessa sii wajjiin turuuf fedhii qabaa?	1.Eeyyee 2. Lakki	
116	Haati/abbaan abba warraa keessani iddoo turtii hadholii sii wajjiin turuuf eeyyamamoo dha?	1.Eeyyee 2. Lakki	
117	Ollaan keessan iddoo turtii haadholii akka turtan isin deeggaru?	1.Eeyyee 2. Lakki	
118	Aadaan kee iddoo turtii haadholii akka fayyadamtu si deeggaraa?	1.Eeyyee 2. Lakki	
119	Ijoollee biroo kunuunsa barbaadan mana keessanitti qabduu?	1.Eeyyee 2. Lakki	
120	Yeroo iddoo turtii haadholii turtanitti namaa isin wajjiin turuu fiduu dandeessu (hayyamni jiraa)?	1.Eeyyee 2. Lakki	
121	Namaa manatti hojii siif hojjatuu maatii keessa ni qabda?	1.Eeyyee 2. Lakki	
122	Namaa sii wajjiin turuu argachuu dandeessa	1.Eeyyee 2. Lakk	
123	Garaa dhaabbata fayyaa maaliin dhuftaa?	1.Konkolaataa 2. Fardaan<br>3. Miilaan 4. Kan biroo	
124	Dhaabbata fayyaa dhihoo jiruu dhaqqabuuf miilaan yeroo hangamii fudhata?	Daqiiqaa (________)	
125	Yoo konkolaataa fayyadamtan: km meeqaa ta’aa?	(________) Km	

Kutaa II Amalootaa walhormaataa

lakk	Gaaffilee	Ramaddiiwwan	Irra darbuu
201	Umuriin ga'eela jalqabaa meeqaa ?	(-------)	
202	Umurii yeroo ulfa jalqabaa meeqa?	(-------)	
203	Yeroo meeqa deesse?	1.Yeroo tokkoo 2. Lamaa fi isaa olii	Deebiin kee yoo 1 ta'ee,208 deemi
204	Yoo 2 fi isaa ol deesse, daa'ima ammaa deessee fi kan duraa gidduu garaagarumma wagga meeqaatti jira (adda fageenya dhalootaa)	(___) waggaa	
205	Daa'imaa xiqqichaa (maandhaa) duraa jiruu eessatti deessee?	1. Dhaabbata fayyaa<br>2. Manaa 3. Kan biroo ibsi	
206	Yeroo ulfaa fi da'umsaa rakkoon isin mudatee jiraa?	1. Eeyyee 2. Lakki	Yoo lakki ta'e gara 210 deemi
207	Yoo deebiin kee eeyyee ta'e: rakkoon gosa akkamii si mudate (deebiin baay'een ni danda'ama).	1.Dhiigaa dahumsa duraa<br>2. Ciniinsuu yeroo malee<br>3. Ciniinsuun harkifachuu<br>4.Dhiigni da'umsa boodaa baay'achuu<br>5.Ukkaamamu daa'imaa<br>6. Preeclampsia & eclampsia<br>7. Daa'imni du'ee dhalachuu<br>8. Kan biroo ibsi	
208	Ulfa kanaaf kunuunsa dahumsa duraa argatteertaa?	1. Eeyyee 2. Lakki	Yoo deebiin kee lakkii ta'ee garaa 301 deemii
209	Yoo deebiin kee eeyyee ta'e: yoom eegalte? Deebii kee torbaniin kaa'i	(____)	
210	Yeroo ulfaa kana yeroo meeqa kunuunsa dahumsa duraa argatte?	(___)	
211	Yeroo daawwannaa ANC keessanitti waa'ee iddoo turtii haadholee odeeffannoo fi gorsa argatteerta?	1. Eeyyee 2. Lakki	

Kutaa III: **Beekumsa iddoo turtii hadhoolii fi barbaachisumma isaa kan haadholiin beekan**

lakk	Gaaffilee	Ramaddiiwwaan	Irraa
301	Waa'ee iddoo turtii haadholii fi barbaachisummaa isaa beektaa?	1.Eeyyee 2. Lakki	Yoo lakki ta'e gara303 deemi
302	Yoo deebiin lakkoofsa gaaffii 301 eeyyee ta’e, barbaachisummaa isaa tarreessi (Deebiin hedduun ni danda’amaa)	1.Du’a sababa rakkoo walxaxaa ulfaatiindhufuu hambisaa<br>2.Haadholiin kunuunsa da’uumsaan boodaa yeroon argachuu danda’u<br>3.Odeefannoo fayyaa hedduu argatu<br>4. Sodaa malee nagaadhaan deessi<br>5. Kan biroo ibsii.	
303	Mallattoolee balaa ciccimoo yeroo ulfaa beektaa?	1.Eeyyee 2. Lakki	Yoo lakki ta'e gara 305 deemii
304	Lakkoofsi gaaffii 303f deebii kennite eeyyee yoo ta’e , mallattoolee beektu tarreessii(Deebiin baay’ee ta’uu danda’a)	1.Qaamaa saalaa keessa dhiigni dhangala’uu<br>2. Gagggabsuu/maraammartoo<br>3.Hoo’aa qaamaa cimaa fi ittii dukkanaa;uu<br>4.Hoo’aa qaamaa cimaa fi dadhabbii<br>5. Dhukkubbii baay’ee cimaa<br>6. Hafuuraa baasuu dadhabuu ykn baay’ee harganuu.	
305	Iddoon turtii haadholii daa’ima dhalattuuf faayidaa qabaa?	1.Eeyyee 2. Lakki	Yoo lakki ta'e gara 307 deemii
306	Yoo deebiin gaaffii lakkoofsa305 eeyyee ta’e, tarreesi (Deebiin baay’een ni danda’ama)	1.Sababa wal -xaxaa da’uumsaatiin duutii akka hin mudanne godha<br>2.Kunuunsa da’uumsaan boodaa yeroon argatu<br>3.Talallii yeroon argatu	
307	Hadholiin ulfaa iddoo turtii haadholii yoom akkaa turuu qaban beektaa?		yoo lakkii ta’ee garaa 401 deemi
308	Yoo deebiin gaaffii 307 eeyyee ta’e, yoomii?	Torbaaniin (__)	

Kutaa IV: Ilaalcha haadholiin itti fayyadama iddoo turtii irratti qaban

lakk	Ilaalcha	Cimsee deeggara =5	Waliigala a=4	G/galee ssa=3	Walii hin galu=2	Cimsee walii hin galuu=1
401	Iddoon turtii haadholii fayyaa haadholii fi daa'immaniitiif barbaachisaa dha					
402	Hadholiin ulfaa iddoo turtii haadholiikeessaa turuun gatii qaba.					
403	Dafaniigara iddoo turtii haadholii da'uumsaaf yeroon deemuun qarummaa					
404	Iddoon turtii haadholii sababaafageenyaatiif barfannaa haadholii mudatuu hambisaa					
405	Dubartootni ulfaa abbaa manaa fi ijoollee irraa adda bahuun da'uumsaaf iddoo turtiituruun miidhaa hin qabuu					
406	Iddoon turtii haadholii dubartootni ulfi xaxamaan qunname gargaarsa narsootaa fi deessistootaa olaanoo akkaa argatan taasisaa					
407	Dhaabbatni iddoo turtii haadholii qabuu kan hin qabnee caala fayyaa haadholii fi daa'immaaniitiif bu'aa qabeessa					
408	Da'uumsaaf iddoo turtii haadholii turuun haatii nagaan akkaa deessuu taasisaa					

Kutaa V: Ittii fayyadama MWH

lakk	Gaaffilee	Ramaddiiwwaan	Irraa darbuu
501	Iddoo turtii haadholii ulfaa dhumaa irratti ciniinsuun osoo sii jalaa hin qabin turteerta?	1.Eeyyee 2. Lakki	Yoo lakkii ta'e gara 506 tti darbi
502	Deebiin gaaffii Lakkoofsi 501 eeyyeeyoo ta'e, sababni kee maali?<br>(deebiin baay'een ni danda'ama)	1.Ulfii koo kan duraa wal-xaxaa ture<br>2. Manni koo dhaabbata fayyaa irraa waan fagaatuuf,<br>3 . Duraan waan fayyadameef<br>4.HEF yeroon da'uumsaa koo dhihaachuu waan natti himaniif<br>5. Kan biroo	
503	Iddoon turtii haadholii sittii toleeraa?	1. Eeyyee 2. Lakki	Yoo lakkii ta'ee garaa 505 darbii
504	Yoo deebiin kee gaaffii 503 eeyyee ta'e, maaltu sittii tole? (Deebiin baay'een ni danda'ama).	1. Namni nyaata bilcheessu jiraachu<br>2. Sireen jiraachu<br>3. Bishaan jiraachu<br>4. Mannii fincaani jiraachu<br>5. Nyaatni jiraachu<br>6. Iddoon bal'aa ta'uu	
505	Yoo deebiin kee gaaffii 503 lakki ta'e, maaltu sitti hin tolin? (Deebiin baay'een ni danda'ama)	1. ogeessi haala gaariin tajaajilaa hin kennuu<br>2.Iccitiin hin eegamu<br>3. maatii irraa fagaachu<br>4.kan biroo	
506	Yoo deebiin kee lakkoofsa 501 lakki ta'e, sababni kee maalii? (Deebin baay'een ni danda'ama).	1.Barbaachisummaa isaa waan hin beekneef<br>2.Eeyyamni maatii naaf hin kennamne<br>3.Maatiin manattii deeggarsa koo waan barbaadduuf<br>4. Manii koo buufataa fayyaatti dhihoo waan jiruuf<br>5. Kan biroo (haa ibsamu)	

Kutaa VI: Dhimmoota dhaabbata waliin walqabatan

Lakk	Gaaffilee	Ramaddiiwwan	Irra darbuu
601	Iddoon turtii haadholii iddoo nyaata ittibilcheessan qabaa?	1.Eeyyee 2. Lakki	Yoo lakki ta'e gara 603 darbi
602	Yoo deebiin gaaffii Lakk.601 eeyyee ta’ee meeshaaleen nyaata ittii bilcheessanii f nyaatani jiraa?	1.Eeyyee 2. Lakki	
603	Iddoon turtii haadholii mana fincaanii ofii qabaa?	1.Eeyyee 2 . Lakk	
604	Iddoo turtii haadholii keessa kutaan ciisichaa jiraa?	1.Eeyyee 2. Lakki	Yoo lakki ta’ee garaa 607 darbi
605	Yoo gaaffiin 604 eeyyee ta’ee kutaa tokkoo keessaa haadholii hammamittirafaa?	------------	
606	Namaa iddoo turtii hadholii dubartii ulfaa wajjiin turuuf bakki ciisichaa jiraa?	1.Eeyyee 2. Lakki	
607	Golgii iccitii (privacy screen) jiraa?	1.Eeyyee 2. Lakki	
608	Ijoollee gara iddoo turtii haadholii fiduun ni danda'amaa?	1.Eeyyee 2. Lakki	
609	Maatiin iddoo turtiitti sii daawwachuu danda'uu?	1.Eeyyee 2. Lakki	
610	Sagantaan sirna buna aadaa ni jiraa?	1.Eeyyee 2. Lakki	
611	Nyaata aadaa naannoo bu'ureeffate ni argatu?	1.Eeyyee 2. Lakki	
612	Erga daa’imni dhalatee booda iddoo turtii kanattii sirnaa aadaa gaggeeffachu dandeessuu?	1.Eeyyee 2. Lakki	

Kutaa VII Ilaalcha Haadholiin CRC ogeessaa fayyaa irratti qaban

Lakk	Ilaalcha CRC ogeessa Fayyaa	Cimsee Waliigalaa=5	Waliigala=4	G/galeessa=3	Walii hin galuu=2	Cimsee walii hin galuu=1
701	Ogeeyyiin fayyaa haadholii iddoo turtii jiran ni kabaju					
702	Ogeeyyiin fayyaa haadholii iddoo turtii jiraniif kunuunsa gaarii ni kennu					
703	Ogeeyyiin fayyaa haadholii gara laaffinnaadhaan tajaajilu					

Galatoomaa!!

Qajeelfamaa KII
Odeeffannoo hirmaattootaa
Kebele______Umrii____Amantii_____Sadarkaa barnootaa_______ Ga’eela___Hojii_____Galii ji’aa______Guyyaa af-gaaffii_________Yeroo ittii jalqabee____yeroo ittii xummurame_____ maqaa gaafataa_________

Lakk	Gaaffilee KII
1.	Rakkoon fayyaa haadholii naannoo kanaa maalii___ (fkn kenniif) du’a haadha, da’umsamanaa, kanneen biro
2.	Dubartoonni naannoo kanaa eessatti da'uu? Fkn, Mana, dhaabbata fayyaa fi kanneenbiroo
3.	Yoo mana ta’e maaliif mana filatu jettanii yaaddu? Fkn, Qulqullina tajaajila fayyaa,TBA, fageenyaa fi geejjibaa
4.	Ummanni naannoo kanaa iddoon turtii haadholii ulfaa dhaabbata fayyaa keessa jiraachuu ni beekaa?
5.	Odeeffannoo iddoo turtii haadholii uummatni eessaa argataa?
6.	Dubartootni iddoo turtii haadholii fayyadamaa jiruu? Haalii ittii fayyadamaa isaa akkaa yaadaa keetiitti maal fakkaata?
7.	Iddoo turtii haadholii ittii fufsisuuf hirmaannaan uummataa maal fakkaata? Qarshii, midhaan
8.	Akka ilaalcha keessaniitti, wantootni itti fayyadama iddoo turtii haadholii naannoo kanaa irratti dhiibbaa geessisan maali jettanii yaaddu? Fkn Aadaa, qulqullina tajaajilaa, fageenya
9	Komiin dubartootni iddoo turtii haadholii irratti qaban kan dhageessee jira? yoo jiraatee tarreessi__fkn, Nyaata, iccitii, hanqina meeshaalee bu’uuraa fi kanneen biroo
10.	Itti fayyadama iddoo turtii haadholii fooyyessuuf maaltu hojjetamu qabaa jettanii yaaddu? fkn, Akka mootummaatti? Michoota, hawaasa, dhaabbata fayyaa
11.	Yaada waliigalaa itti dabaluu barbaaddan yoo jiraate ibsaa
